# Implantation of a Resynchronization Implantable Cardioverter Defibrillator in a Patient with Persistent Left Superior Vena Cava

**Published:** 2007-10-22

**Authors:** Dante Antonelli, Nahum Adam Freedberg, Alexander Feldman

**Affiliations:** Dept. of Cardiology, Ha Emek Medical Center, 18101 Afula, Israel

**Keywords:** Left Persistent Vena Cava, Implantable Cardioverter Defibrillator, Resynchronization therapy

## Abstract

Implantation of resynchronization implantable cardioverter defibrillator was performed in a patient with persistent left superior vena cava. A dual coil defibrillation lead was inserted in the right ventricle apex via a small innominate vein. Left ventricular and atrial leads were implanted through persistent left superior vena cava. Left ventricular lead was easily implanted into the postero lateral vein. Pacing thresholds and sensing values were excellent and remained stable at 18 months follow-up.

Presence of persistent left superior vena cava generally makes transvenous lead implantation difficult. However when a favorable coronary sinus anatomy is also present, it may facilitate left ventricular lead positioning in the coronary sinus branches.

## Introduction

Persistent left superior vena cava (PLSVC) is a congenital anomaly that is present in 0.5% of the general population [1]. In 68% of cases an innominate vein bridges the two superior venae cavae [2]. It usually drains into the proximal segment of a dilated coronary sinus (CS) and from there on towards the right atrium (RA). Generally it remains asymptomatic and is an unexpected finding during pacing lead implantation. We report a case of resynchronization implantable cardioverter defibrillator (CRT-D) implantation in a patient with PLSVC.

## Case Report

A 54 year old man suffering from congestive heart failure (NYHA class II) and dilated cardiomyopathy was admitted because of syncope. Baseline ECG revealed sinus rhythm, QRS complex duration 190 ms and LBBB. Echocardiography showed an enlarged left ventricle and ejection fraction of 25%. Coronary angiography documented normal coronary arteries and a vascular structure, suspected to be PLSVC, during the venous phase. Implantation of a (CRT-D) was planned.

**Figure 1:**
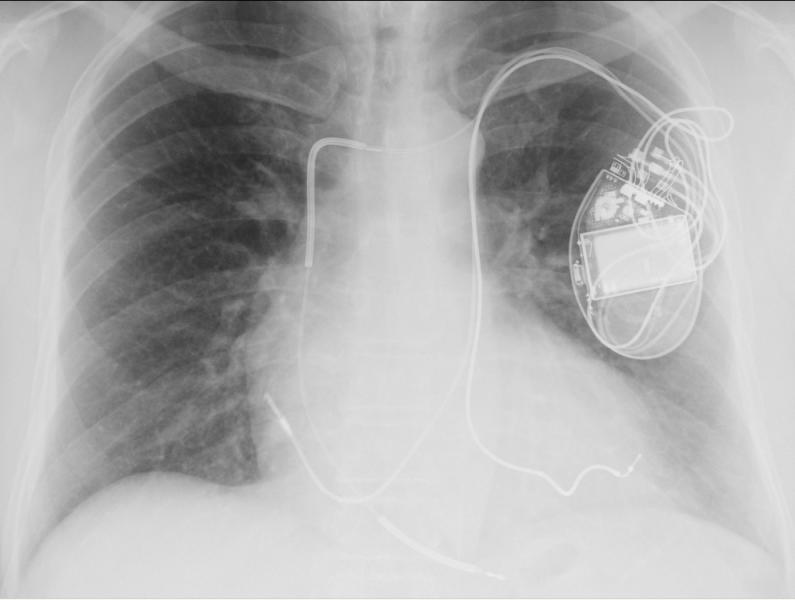
AP projection: the left ventricular lead runs via the PLSVC - coronary sinus to the postero lateral vein.

Cannulation of the left subclavian vein was performed. Venography confirmed a PLSVC, draining into the coronary sinus next to the origin of the postero lateral vein, and the existence of a small innominate vein, draining into the right superior vena cava. A screw-in dual coil lead (Endotak Reliance™, model 0165, Guidant Corp., St. Paul, MN, USA) was introduced in the apex of the right ventricle (RV) via the innominate vein. Stimulation threshold measured 0.5 V at 0.5ms with an impedance of 1185 Ω, R wave amplitude was 15.3mV with slew rate of > 4 V/s, high-voltage shock impedance was 47 Ω. The left ventricular (LV) lead (Easytrak® 3, model 4525, Guidant Corp.) was easily advanced through the PLSVC into the postero lateral vein (Figure 1). Its stimulation threshold was 0.7V at 0.5 ms, R wave amplitude 13.6 mV, slew rate > 4V/s and impedance 1059 Ω.

An attempt to introduce a second lead (atrial lead) through the innominate vein failed because of the small vein diameter. Subsequently a screw-in, 59 cm length lead (Flextend®, model 4088, Guidant Corp.) was successfully advanced into the right atrium via PLSVC-coronary sinus and was screwed in its anterior wall. Stimulation threshold measured 1.5 V at 0.5 ms, P wave amplitude 2.5 mV, slew rate 0.5 V/s and impedance 832 Ω. The leads were connected to a CRT-D device (Contak® Renewal™ 4HE, model H199, Guidant Corp.), implanted in the left pectoral area. The defibrillation threshold test was not performed during the implant session. The following day, ventricular fibrillation induced by a T wave shock was successfully defibrillated with 21J. During 18 months follow-up atrial, RV and LV sensing and stimulation thresholds were excellent.

## Discussion

PLSVC limits lead handling during the implantation because of the altered venous course. Its presence makes transvenous leads implantation challenging or even impossible in some cases [3]. The major difficulty is RV lead implantation because the tip of the lead tends to be deflected away from the tricuspid orifice when entering the RA. In our patient a small innominate vein was present and allowed a conventional RV lead implantation, but no other leads could be advanced through it. LV lead was introduced through the PLSVC into the CS and was implanted in the postero lateral coronary vein  that presented an easy accessibility of its ostium. Easy "downstream" LV lead positioning been reported by others also [4-7]. However when acute angles are encountered at the branches of CS ostia lead positioning may be challenging also during "downstream" catheterization of CS [8-10].

The difficulty to intubate the CS is still one of the reasons for failing to implant a biventricular pacing system. The presence of PLSVC avoids the need of CS intubation and, together with a favorable CS anatomy, may facilitate LV lead positioning in the CS branches.
